# Accuracy Assessment of Nondispersive Optical Perturbative Models through Capacity Analysis

**DOI:** 10.3390/e21080760

**Published:** 2019-08-05

**Authors:** Kamran Keykhosravi, Giuseppe Durisi, Erik Agrell

**Affiliations:** Department of Electrical Engineering, Chalmers University of Technology, 41296 Gothenburg, Sweden

**Keywords:** achievable rate, channel capacity, information theory, nonlinear channel, optical fiber

## Abstract

A number of simplified models, based on perturbation theory, have been proposed for the fiber-optical channel and have been extensively used in the literature. Although these models are mainly developed for the low-power regime, they are used at moderate or high powers as well. It remains unclear to what extent the capacity of these models is affected by the simplifying assumptions under which they are derived. In this paper, we consider single-channel data transmission based on three continuous-time optical models: (i) a regular perturbative channel, (ii) a logarithmic perturbative channel, and (iii) the stochastic nonlinear Schrödinger (NLS) channel. To obtain analytically tractable discrete-time models, we consider zero-dispersion fibers and a sampling receiver. We investigate the per-sample capacity of these models. Specifically, (i) we establish tight bounds on the capacity of the regular perturbative channel; (ii) we obtain the capacity of the logarithmic perturbative channel; and (iii) we present a novel upper bound on the capacity of the zero-dispersion NLS channel. Our results illustrate that the capacity of these models departs from each other at high powers because these models yield different capacity pre-logs. Since all three models are based on the same physical channel, our results highlight that care must be exercised in using simplified channel models in the high-power regime.

## 1. Introduction

The vast majority of the global Internet traffic is conveyed through fiber-optical networks, which form the backbone of our information society. To cope with the growing data demand, the fiber-optical networks have evolved from regenerated direct-detection systems to coherent wavelength division multiplexing (WDM) ones. Newly emerging bandwidth-hungry services, like Internet-of-Things (IoT) applications and cloud processing, require even higher data rates. Motivated by this ever-growing demand, an increasing attention has been devoted in recent years to the analysis of the capacity of the fiber-optical channel.

Finding the capacity of the fiber-optical channel that is governed by the stochastic nonlinear Schrödinger (NLS) equation ([1], Equation (1)), which captures the effects of Kerr nonlinearity, chromatic dispersion, and amplification noise, remains an open problem. An information-theoretic analysis of the NLS channel is cumbersome because of a complicated signal–noise interaction caused by the interplay between the nonlinearity and the dispersion [2]. In general, capacity analyses of optical fibers are performed either by considering simplified channels, or by evaluating mismatched decoding lower bounds [3] via simulations (see [4] and ([5], Sec. I) for excellent literature reviews). Lower bounds based on the mismatch-decoding framework go to zero after reaching a maximum (see, for example, [2,6,7,8,9]). Capacity lower bounds with a similar behavior are also reported in [10]. In [11], it has been shown that the maximum value of a capacity lower bound can be increased by increasing fiber dispersion, which mitigates the effects of nonlinearity. To establish a capacity upper bound, Kramer et al. [12] used the split-step Fourier (SSF) method, which is a standard approach to solve the NLS equation numerically ([13], Sec. 2.4.1), to derive a discrete-time channel model. They proved that the capacity of this discrete-time model is upper-bounded by that of an equivalent additive white Gaussian noise (AWGN) channel. In contrast to the available lower bounds, which fall to zero or saturate at high powers, this upper bound, which is the only one available for a realistic fiber channel model, grows unboundedly.

Since the information-theoretic analysis of the NLS channel is difficult, to approximate capacity, one can resort to simplified models, a number of which have been studied in the literature (see [14] and references therein for a recent review). Two approaches to obtain such models are to use the regular perturbation or the logarithmic perturbation methods. In the former, the effects of nonlinearity are captured by an additive perturbative term [15,16]. This approach yields a discrete-time channel with input–output relation $y_{l}=x_{l}+\Delta x_{l}+n_{l}$ ([14], Equation (5)), where $x_{l}$ and $y_{l}$ are the transmitted and the received symbols, respectively; $n_{l}$ is the amplification noise; and $\Delta x_{l}$ is the perturbative nonlinear distortion. This model holds under the simplifying assumption that both the nonlinearity and the signal–noise interaction are weak, which is reasonable only at low power.

Regular perturbative fiber-optical channel models, with or without memory, have been extensively investigated in the literature. In [17], a first-order perturbative model for WDM systems with arbitrary filtering and sampling demodulation, and coherent detection is proposed. The accuracy of the model is assessed by comparing the value of a mismatch-decoding lower bound, which is derived analytically based on the perturbative model, with simulation results over a realistic fiber-optical channel. A good agreement at all power levels is observed. The capacity of a perturbative multiple-access channel is studied in [18]. It is shown that the nonlinear crosstalk between channels does not affect the capacity region when the information from all the channels is optimally used at each detector. However, if joint processing is not possible (it is typically computationally demanding [19]), the channel capacity is limited by the inter-channel distortion.

Another class of simplified models, which are equivalent to the regular perturbative ones up to a first-order linearization, is that of logarithmic perturbative models, where the nonlinear distortion term $\Delta x_{l}$ is modeled as a phase shift. This yields a discrete-time channel with input–output relation $y_{l}=x_{l}e^{j\Delta x_{l}}+n_{l}$ ([14], Equation (7)). In [5], a single-span optical channel model for a two-user WDM transmission system is developed from a coupled NLS equation, neglecting the dispersion effects within the WDM bands. The channel model in [5] resembles the perturbative logarithmic models. The authors study the capacity region of this channel in the high-power regime. It is shown that the capacity pre-log pair (1,1) is achievable, where the capacity pre-log is defined as the asymptotic limit of C/logP for P→∞, where *P* is the input power and C is the capacity.

Despite the fact that the aforementioned simplified channels are valid in the low-power regime, these models are often used also in the moderate- and high-power regimes. Currently, it is unclear to what extent the simplifications used to obtain these models influence the capacity at high powers. To find out, we study the capacity of two single-channel memoryless perturbative models, namely, a *regular perturbative channel (RPC)*, and a *logarithmic perturbative channel (LPC)*. To assess accuracy of these two perturbative models, we also investigate the per-sample capacity of a *memoryless NLS channel (MNC)*.

To enable an information-theoretic analysis of the fiber-optical channel, we deploy two common assumptions on the channel model. First, the dispersion is set to zero and second, a sampling receiver is used to obtain discrete-time models from continuous-time channels. These two assumptions were first applied to the NLS equation in [1] to obtain an analytically tractable channel model. This channel model was developed also in [20,21,22] using different methods. In this paper, we refer to this model as MNC.

In [21], a lower bound on the per-sample capacity of the memoryless NLS channel is derived, which proves that the capacity goes to infinity with power. In [22], the capacity of the same channel is evaluated numerically. Furthermore, it is shown that the capacity pre-log is 1/2. Approximations of the capacity and optimal input distribution in the intermediate power range are derived in [23,24]. These results are extended to a channel with a more realistic receiver than the sampling one in [25]. The only known nonasymptotic upper bound on the capacity of this channel is log(1+SNR) (bits per channel use) [12], where SNR is the signal-to-noise ratio. This upper bound holds also for the general case of nonzero dispersion.

The novel contributions of this paper are as follows. First, we tightly bound the capacity of the RPC model and prove that its capacity pre-log is 3. Second, the capacity of the LPC is readily shown to be the same as that of an AWGN channel with the same input and noise power. Hence, the capacity pre-log of the LPC is 1. Third, we establish a novel upper bound on the capacity of the MNC (first presented in the conference version of this manuscript [26]). Our upper bound improves the previously known upper bound [12] on the capacity of this channel significantly and, together with the proposed lower bound, allows one to characterize the capacity of the MNC accurately.

Although all three models represent the same physical optical channel, their capacities behave very differently in the high-power regime. This result highlights the profound impact of the simplifying assumptions on the capacity at high powers, and indicates that care should be taken in translating the results obtained based on these models into guidelines for system design.

The rest of this paper is organized as follows. In Section 2, we introduce the three channel models. In Section 3, we present upper and lower bounds on the capacity of these channels and establish the capacity pre-log of the perturbative models. Numerical results are provided in Section 4. We conclude the paper in Section 5. The proofs of all theorems are given in the appendices.

*Notation:* Random quantities are denoted by boldface letters. We use $\mathrm{CN}\left(0,\sigma^{2}\right)$ to denote the complex zero-mean circularly symmetric Gaussian distribution with variance $\sigma^{2}$. We write $R\left(x\right)$, |x|, and *x* to denote the real part, the absolute value, and the phase of a complex number *x*. All logarithms are in base two. The mutual information between two random variables x and y is denoted by I(x;y). The entropy and differential entropy are denoted by H(·) and h(·), respectively. Finally, we use * for the convolution operator.

## 2. Channel Models

The fiber-optical channel is well-modeled by the NLS equation, which describes the propagation of a complex baseband electromagnetic field through a lossy single-mode fiber as

(1) $$\frac{\partial a}{\partial z}+\frac{\alpha -g}{2}a+j\frac{\beta_{2}}{2}\frac{\partial^{2}a}{\partial t^{2}}-j\gamma {\left|a\right|}^{2}a=n.$$

Here, a=a(z,t) is the complex baseband signal at time *t* and location *z*. The parameter γ is the nonlinear coefficient, $\beta_{2}$ is the group-velocity dispersion parameter, α is the attenuation constant, g=g(z) is the gain profile of the amplifier, and n=n(z,t) is the Gaussian amplification noise, which is bandlimited because of the inline channel filters. The third term on the left-hand side of (1) is responsible for the channel memory and the fourth term for the channel nonlinearity.

To compensate for the fiber losses, two types of signal amplification can be deployed, namely, distributed and lumped amplification. The former method compensates for the fiber loss continuously along the fiber, whereas the latter method boosts the signal power by dividing the fiber into several spans and using an optical amplifier at the end of each span. With distributed amplification, which we focus on in this paper, the noise can be described by the autocorrelation function [2]

(2) $$E\left[n\left(z,t\right)n^{*}\left(z^{\prime },t^{\prime }\right)\right]=\alpha n_{\mathrm{sp}}h\nu \delta_{W_{N}}\left(t-t^{\prime }\right)\delta \left(z-z^{\prime }\right).$$

Here, $n_{\mathrm{sp}}$ is the spontaneous emission factor, *h* is Planck’s constant, and ν is the optical carrier frequency. In addition, δ(·) is the Dirac delta function and $\delta_{W_{N}}\left(x\right)=W_{N}\mathrm{sinc}\left(W_{N}x\right)$, where $W_{N}$ is the noise bandwidth. In this paper, we shall focus on the ideal distributed-amplification case g(z)=α.

We use a sampling receiver to go from continuous-time channels to discrete-time ones. A comprehensive description of the sampling receiver and of the induced discrete-time channel is provided in ([22], Section III). Here, we review some crucial elements of this description for completeness. Assume that a signal a(0,t), which is band-limited to $W_{0}$ hertz, is transmitted through a zero-dispersion NLS channel ((1) with $\beta_{2}=0$) in the time interval [0,T]. Because of nonlinearity, the bandwidth of the received signal a(L,t) may be larger than that of a(0,t). To avoid signal distortion by the inline filters, we assume that $W_{0}$ is set such that a(z,t) is band-limited to $W_{N}$ hertz for 0≤z≤L. Since $W_{0}\leq W_{N}$, assuming $W_{N}T\gg 1$, both the transmitted and the received signal can be represented by $2W_{N}T$ equispaced samples. The transmitter encodes data into subsets of these samples of cardinality $2W_{0}T$, referred to as the principal samples. At the receiver, demodulation is performed by sampling a(L,t) at instances corresponding to the principal samples. This results in $2W_{0}T$ parallel independent discrete-time channels that have the same input–output relation.

The sampling receiver has a number of shortcomings [27] and using it should be considered a simplification. The resulting discrete-time model is used extensively in the literature (see, for example, [1,20,21,22,28,29]), since it makes analytical calculation possible. In this paper, we apply the sampling receiver not only to the memoryless NLS channel but also to the memoryless perturbative models.

Next, we review two perturbative channel models that are used in the literature to approximate the solution of the NLS Equation (1). Among the multiple variations of perturbative models available in the literature, we use the ones proposed in [30]. For both perturbative models, first continuous-time dispersive models are introduced, and then memoryless discrete-time channels are developed by assuming that $\beta_{2}=0$ and by using a sampling receiver. Finally, we introduce the MNC model, which is derived from (1) under the two above-mentioned assumptions.

*Regular perturbative channel (RPC):* Let $a^{\mathrm{li}}\left(z,t\right)$ be the solution of the linear noiseless NLS equation (Equation (1) with n(z,t)=0 and γ=0). It can be computed as $a^{\mathrm{li}}\left(z,t\right)=a\left(0,t\right)*h\left(z,t\right)$, where $h\left(z,t\right)=F^{-1}\left\{\exp\left(j\beta_{2}\omega^{2}z/2\right)\right\}$ and $F^{-1}\left(\cdot \right)$ denotes the inverse Fourier transform. In the regular perturbation method, the output of the noiseless NLS channel (Equation (1) with n=0) is approximated as

(3) $$a\left(L,t\right)=a^{\mathrm{li}}\left(L,t\right)+\Delta a\left(L,t\right).$$

Here, *L* is the fiber length and Δa(z,t) is the nonlinear perturbation term. If now the model is expanded to include amplification noise as an additive noise component, neglecting signal–noise interactions, then the accumulated amplification noise

(4) $$w\left(L,t\right)=\int_{0}^{L}n\left(z,t\right)\;dz$$

can be added to the signal at the receiver to obtain the channel model ([14], Equation (5))

(5) $$a\left(L,t\right)=a^{\mathrm{li}}\left(L,t\right)+\Delta a\left(L,t\right)+w\left(L,t\right).$$

The first-order approximation of Δa(L,t) is ([30], Equation (13))

(6) $$\Delta a\left(L,t\right)=j\gamma \int_{0}^{L}\left[{\left|a^{\mathrm{li}}\left(\zeta ,t\right)\right|}^{2}a^{\mathrm{li}}\left(\zeta ,t\right)\right]*h\left(L-\zeta ,t\right)\;d\zeta ,$$

where the convolution is over the time variable. (Using higher-order nonlinear terms improves the accuracy of the regular perturbative channels. However, in this paper, we focus only on the channel model based on the first-order approximation, which is commonly used in the literature.) Neglecting dispersion (i.e., setting $\beta_{2}=0$), we have h(z,t)=δ(t) and $a^{\mathrm{li}}\left(\zeta ,t\right)=a\left(0,t\right)$. Using this in (6), and then substituting (6) into (5), we obtain

(7) $$a\left(L,t\right)=a\left(0,t\right)+{jL\gamma |a\left(0,t\right)|}^{2}a\left(0,t\right)+w\left(L,t\right).$$

Finally, by deploying sampling receiver, we obtain from (7) the discrete-time channel model

(8) $$y=x+{j\eta |x|}^{2}x+n.$$

Here, $n∼\mathrm{CN}\left(0,P_{N}\right)$,

(9) $$P_{N}=2\alpha n_{\mathrm{sp}}h\nu LW_{N}$$

is the total noise power, and

(10) η=γL.

We refer to (8) as the RPC.

*Logarithmic perturbative channel (LPC):* Another method for approximating the solution of the NLS Equation (1) is to use logarithmic perturbation. With this method, the output signal is approximated as ([14], Equation (7))

(11) $$a\left(L,t\right)=a\left(0,t\right)\exp\left(j\Delta \theta (L,t)\right)+w\left(L,t\right),$$

where w(L,t) is the same noise term as in (5)–(4). The first-order approximation of Δθ(L,t) is ([30], Equation (19))

(12) $$\Delta \theta \left(L,t\right)=\frac{\gamma }{a^{\mathrm{li}}\left(L,t\right)}\int_{0}^{L}\left[{\left|a^{\mathrm{li}}\left(\zeta ,t\right)\right|}^{2}a^{\mathrm{li}}\left(\zeta ,t\right)\right]*h\left(L-\zeta ,t\right)\;d\zeta .$$

Under the zero-dispersion assumption ($\beta_{2}=0$), we have h(z,t)=δ(t) and $a^{\mathrm{li}}\left(\zeta ,t\right)=a\left(0,t\right)$. Using this in (12), and then substituting (12) into (11), we obtain

(13) $$a\left(L,t\right)=a\left(0,t\right)e^{{j\gamma L|a\left(0,t\right)|}^{2}}+w\left(L,t\right).$$

Finally, by sampling the output signal, the discrete-time channel

(14) $$y=xe^{{j\eta |x|}^{2}}+n$$

is obtained, where $n∼\mathrm{CN}\left(0,P_{N}\right)$, $P_{N}$ is given in (9), and η is defined in (10). We note that the channels (8) and (14) are equal up to a first-order linearization, which is accurate in the low-power regime. Furthermore, one may also obtain the model in (13) by solving (1) for $\beta_{2}=0$, n=0, and g=α and by adding the noise at the receiver.

*Memoryless NLS Channel (MNC):* Here, we shall study the underlying NLS channel in (1) under the assumptions that $\beta_{2}=0$ and that a sampling receiver is used to obtain a discrete-time channel. Let $r_{0}$ and $\theta_{0}$ be the amplitude and the phase of a transmitted symbol x, and let r and θ be those of the received samples y. The discrete-time channel input–output relation can be described by the conditional probability density function (pdf) ([20], Ch. 5) (see also ([28], Sec. II))

(15) $$\begin{array}{c} f_{r,\theta |r_{0},\theta_{0}}\left(r,\theta |r_{0},\theta_{0}\right)=\frac{f_{r|r_{0}}\left(r|r_{0}\right)}{2\pi }+\frac{1}{\pi }\sum_{m=1}^{\infty }R\left(C_{m}\left(r\right)e^{-jm(\theta -\theta_{0})}\right). \end{array}$$

The conditional pdf $f_{r|r_{0}}\left(r|r_{0}\right)$ and the Fourier coefficients $C_{m}\left(r\right)$ in (15) are given by

(16) $$\begin{array}{cc} f_{r|r_{0}}\left(r|r_{0}\right) & =\frac{2r}{P_{N}}\exp\left(-\frac{r^{2}+r_{0}^{2}}{P_{N}}\right)I_{0}\left(\frac{2rr_{0}}{P_{N}}\right), \end{array}$$

(17) $$\begin{array}{cc} C_{m}\left(r\right) & =2r\nu_{m}\exp\left(-\left(r^{2}+r_{0}^{2}\right)\nu_{m}\cos x_{m}\right)I_{m}\left(2rr_{0}\nu_{m}\right). \end{array}$$

Here, $I_{m}\left(\cdot \right)$ denotes the *m*th order modified Bessel function of the first kind, and

(18) $$\begin{array}{cc} x_{m} & ={\left(\frac{2jm\gamma r_{0}^{2}P_{N}L}{2r_{0}^{2}+P_{N}}\right)}^{1/2}, \end{array}$$

(19) $$\begin{array}{cc} \nu_{m} & =\frac{x_{m}}{P_{N}\sin x_{m}}. \end{array}$$

The complex square root in (18) is a two-valued function, but both choices give the same values of $\nu_{m}$ and $C_{m}\left(r\right)$.

In the next section, we study the capacity of the channel models given in (8), (14), and (15). Since all of these models are memoryless, their capacities under a power constraint *P* are given by

(20) C=supI(x;y),

where the supremum is over all complex probability distributions of x that satisfy the average-power constraint

(21) $$E\left[{\left|x\right|}^{2}\right]\leq P.$$

## 3. Analytical Results

In this section, we study the capacity of the RPC, the LPC, and the MNC models. All these models are based on the same fiber-optical channel and share the same set of parameters. Bounds on the capacity of the RPC in (8) are provided in Theorems 1–3. Specifically, in Theorem 1, we establish a closed-form lower bound, which, together with the upper bound provided in Theorem 2, tightly bounds capacity (see Section 4). A different upper bound is provided in Theorem 3. Numerical evidence suggests that this alternative bound is less tight than the one provided in Theorem 2 (see Section 4). However, this alternative bound has a simple analytical form, which makes it easier to characterize it asymptotically. By using the bounds derived in Theorems 1 and 3, we prove that the capacity pre-log of the RPC is 3. In Theorem 4, we derive the capacity of the LPC in (14) and show that it coincides with the capacity of an equivalent AWGN channel. Hence, the capacity pre-log is 1. Theorem 4 is rather trivial and we present it in this section only for completeness. Finally, in Theorem 5, we provide an upper bound on the capacity of the MNC in (15), which improves the previous known upper bound [12] significantly, and, together with a proposed capacity lower bound, yields a tight characterization of capacity (see Section 4).

### 3.1. Capacity Analysis of the RPC

**Theorem** **1.** The capacity $C_{\mathrm{RPC}}$ of the RPC in (8) is lower-bounded by

(22) $$\begin{array}{cc} C_{\mathrm{RPC}}\geq L_{\mathrm{RPC}}\left(P\right) & =\max_{\lambda }\left\{\log\left(\frac{\lambda^{2}+6\eta^{2}}{\lambda^{3}P_{N}}\;e^{\frac{12\eta^{2}}{\lambda^{2}+6\eta^{2}}}+1\right)\right\}, \end{array}$$

where λ is positive and satisfies the constraint

(23) $$\begin{array}{c} \frac{18\eta^{2}+\lambda^{2}}{\lambda \left(6\eta^{2}+\lambda^{2}\right)}\leq P. \end{array}$$

Furthermore, the maximum in (22) is achieved by the unique real solution of the equation

(24) $$\begin{array}{cc} P\lambda^{3} & -\lambda^{2}+6P\eta^{2}\lambda -18\eta^{2}=0. \end{array}$$

**Proof.**  See Appendix A. □

**Theorem** **2.** The capacity of the RPC in (8) is upper-bounded by

(25) $$\begin{array}{cc} C_{\mathrm{RPC}} & \leq U_{\mathrm{RPC}}\left(P\right)\\ & =\min_{\mu >0,\;\lambda >0}\left\{\log\frac{\mu^{2}+6\eta^{2}}{\mu^{3}eP_{N}}+\lambda +\max_{s>0}\left\{\mu E\left[q\left({\left|y\right|}^{2}\right)||x{|}^{2}=s\right]\log e-\lambda \frac{s+P_{N}}{P+P_{N}}\right\}\right\}. \end{array}$$

Here, $q\left(x\right)=g^{-1}\left(x\right)$, where $g\left(x\right)=x+\eta^{2}x^{3}$.

**Proof.**  See Appendix B. □

Note that, given ${\left|x\right|}^{2}=s$, the random variable ${2|y|}^{2}/P_{N}$ is conditionally distributed as a noncentral chi-squared random variable with two degrees of freedom and noncentrality parameter $2\left(s+\eta^{2}s^{3}\right)/P_{N}$. This enables numerical computation of $U_{\mathrm{RPC}}\left(P\right)$.

**Theorem** **3.** The capacity of the RPC in (8) is upper-bounded by

(26) $$\begin{array}{c} C_{\mathrm{RPC}}\leq {\tilde{U}}_{\mathrm{RPC}}\left(P\right)=\min_{\mu >0}\left\{\log\left(\frac{\mu^{2}+6\eta^{2}}{\mu^{3}eP_{N}}\right)+\mu \left(P+B\right)\log e\right\}, \end{array}$$

where

(27) $$\begin{array}{c} B=P_{N}+\frac{\sqrt{\pi P_{N}}}{12^{3/8}\sqrt{(\sqrt{3}-1)\eta }}. \end{array}$$

Furthermore, the minimum in (26) is achieved by the unique real solution of the equation

(28) $$\begin{array}{c} \left(P+B\right)\mu^{3}-\mu^{2}+6\eta^{2}\left(P+B\right)\mu -18\eta^{2}=0. \end{array}$$

**Proof.**  See Appendix C. □

*Pre-log analysis:* By substituting μ=1/P into (26), we see that

(29) $$\begin{array}{c} \lim_{P\to \infty }\left[C_{\mathrm{RPC}}-3\log\left(P\right)\right]\leq \log\left(\frac{6\eta^{2}}{P_{N}}\right). \end{array}$$

Furthermore, since

(30) $$\begin{array}{cc} \frac{18\eta^{2}+\lambda^{2}}{\lambda \left(6\eta^{2}+\lambda^{2}\right)} & \leq \frac{18\eta^{2}+3\lambda^{2}}{\lambda \left(6\eta^{2}+\lambda^{2}\right)} \end{array}$$

(31) $$\begin{array}{c} =\frac{3}{\lambda }, \end{array}$$

we can obtain a valid lower bound on $C_{\mathrm{RPC}}$ by substituting λ=3/P into (22). Doing so, we obtain

(32) $$\begin{array}{c} \lim_{P\to \infty }\left[C_{\mathrm{RPC}}-3\log\left(P\right)\right]\geq \log\left(\frac{2\eta^{2}e^{2}}{9P_{N}}\right). \end{array}$$

It follows from (29) and (32) that the capacity pre-log of the RPC is 3.

### 3.2. Capacity Analysis of the LPC

**Theorem** **4.** The capacity of the LPC in (14) is

(33) $$\begin{array}{c} C_{\mathrm{LPC}}=\log\left(1+\frac{P}{P_{N}}\right). \end{array}$$

**Proof.**  We use the maximum differential entropy lemma ([31], Sec. 2.2) to upper-bound $C_{\mathrm{LPC}}$ by $\log\left(1+P/P_{N}\right)$. Then, we note that we can achieve this upper bound by choosing $x∼\mathrm{CN}\left(0,P\right)$. Alternatively, this theorem can be readily proved by applying a preprocessing step $\tilde{x}={x\exp(-j\eta |x|}^{2})$ to the input and transferring the channel to a linear AWGN channel with the same power constraint, whose capacity is $\log\left(1+P/P_{N}\right)$. □

### 3.3. Capacity Analysis of the MNC

A novel upper bound on the capacity of the MNC in (15) is presented in the following theorem [26].

**Theorem** **5.** The capacity of the MNC in (15) is upper-bounded by

(34)CMNC≤UMNC(P)(35)=minλ>0,α>0αlogP+PNα+logπΓ(α)+λ+maxr0>0{gλ,α(r0,P)},

where Γ(·) denotes the Gamma function and

(36) $$\begin{array}{cc} g_{\lambda ,\alpha }\left(r_{0},P\right) & =\left(\alpha \log e-\lambda \right)\frac{r_{0}^{2}+P_{N}}{P+P_{N}}+\left(1-2\alpha \right)E\left[\log\left(r\right)|r_{0}=r_{0}\right]\\ & \;-h\left(r|r_{0}=r_{0}\right)-h\left(\theta |r,r_{0}=r_{0},\theta_{0}=0\right). \end{array}$$

The upper bound $U_{\mathrm{MNC}}\left(P\right)$ can be calculated numerically using the expression for the conditional pdf $f_{r,\theta |r_{0},\theta_{0}}\left(r,\theta |r_{0},\theta_{0}\right)$ given in (15).

**Proof.**  See Appendix D. □

## 4. Numerical Examples

**Figure 1:**
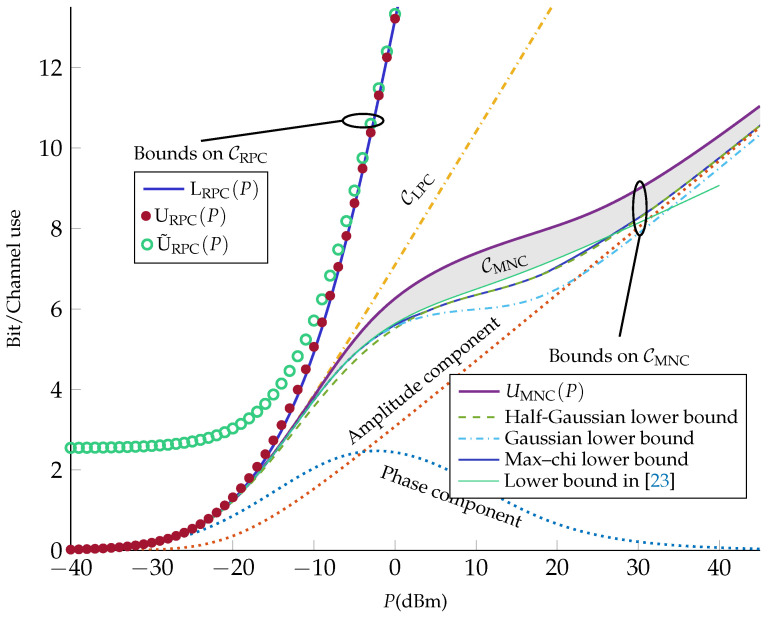
Capacity bounds for the RPC in (8) and the MNC in (15), together with the capacity of the LPC in (14). The amplitude and the phase components of the half-Gaussian lower bound for the MNC are also plotted.

In Figure 1, we evaluate the bounds derived in Section 3 for a fiber-optical channel whose parameters are listed in Table 1. (The channel parameters are the same as in ([22], Table I).) Using (10), we obtain $\eta =6350\;W^{-1}$.

As can be seen from Figure 1, the capacity of the RPC is tightly bounded between the upper bound $U_{\mathrm{RPC}}\left(P\right)$ in (25) and the lower bound $L_{\mathrm{RPC}}\left(P\right)$ in (22). Furthermore, one can observe that although the alternative upper bound ${\tilde{U}}_{\mathrm{RPC}}\left(P\right)$ in (26) is loose at low powers, it becomes tight in the moderate- and high-power regimes.

We also plot the upper bound $U_{\mathrm{MNC}}\left(P\right)$ on the capacity of the MNC. It can be seen that $U_{\mathrm{MNC}}\left(P\right)$ improves substantially on the upper bound given in [12], i.e., the capacity of the corresponding AWGN channel (33) (which coincides with $C_{\mathrm{LPC}}$). As a lower bound on the MNC capacity, we propose the mutual information in (20) with an input x with uniform phase and amplitude $r_{0}$ following a chi distribution with *k* degrees of freedom. Specifically, we set

(37) $$f_{r_{0}}\left(r_{0}\right)=\frac{2r_{0}^{k-1}}{\Gamma (k/2)}{\left(\frac{k}{2P}\right)}^{k/2}\;\;\;\;\exp\left(\frac{-kr_{0}^{2}}{2P}\right),$$

where Γ(·) denotes the gamma function. The parameter *k* is optimized for each power. (Due to the computational complexity, we only considered *k* values from 0.5 to 2.5 in steps of 0.5.) We calculated the bound numerically and include it in Figure 1 (referred to as max–chi lower bound). We also include two lower bounds corresponding to k=1 (with half-Gaussian amplitude distribution, first presented in [22]) and k=2 (with Rayleigh-distributed amplitude, or equivalently, a complex Gaussian input x, first presented in [26]). The max–chi lower bound coincides with these two lower bounds at asymptotically low and high power, and improves slightly thereon at intermediate powers (around 0 dBm), similarly to the numerical bound in [32]. Specifically, at asymptotically low powers, k=2 (Gaussian lower bound) is optimal. This is expected, since the channel is essentially linear at low powers. At high powers, on the other hand, the optimal *k* value approaches 1 (half-Gaussian lower bound), which is consistent with [22], where it has been shown that half-Gaussian amplitude distribution is capacity-achieving for the MNC in the high-power regime. Based on our numerical evaluations, we observed that k=0.5 maximizes the max–chi lower bound (among the set of considered values of *k*) in the power range 18≤P≤32 dBm. Finally, in Figure 1, we plot the lower bound based on the input distribution ([23], Equation (45)). As can be seen, based on our numerical evaluation, this lower bound almost coincides with the max-chi lower bound at low powers (P<−5) and improves on it in the intermediate power range (−5≤P<27 dBm); however, it is suboptimal at high powers (P≥27 dBm).

Figure 1 suggests that $C_{\mathrm{MNC}}$ experiences changes in slope at about 0 and 30 dBm (corresponding to the inflection points at about −10 dBm and 20 dBm). To explain this behavior, we evaluate the phase and the amplitude components of the half-Gaussian lower bound. Specifically, we split the mutual information into two parts as

(38)I(x;y)=I(r0,θ0;r,θ)(39)=I(r0,θ0;r)+I(r0,θ0;θ|r).

The first term in (39) is the amplitude component and the second term is the phase component of the mutual information. These two components are evaluated for the half-Gaussian amplitude distribution and plotted in Figure 1. It can be seen from Figure 1 that the amplitude component is monotonically increasing with power while the phase component goes to zero with power after reaching a maximum. Indeed, at high powers, the phase of the received signal becomes uniformly distributed over [0,2π] and independent of the transmitted signal ([33], Lem. 5). By adding these two components, one obtains a capacity lower bound that changes concavity at two points. The reduction of the capacity slope at intermediate powers is consistent with [23,24], where it is shown that the capacity grows according to loglogP in this regime.

As a final observation, we note that $C_{\mathrm{RPC}}$ diverges from $C_{\mathrm{MNC}}$ at about −15 dBm, whereas $C_{\mathrm{LPC}}$ diverges from $C_{\mathrm{MNC}}$ at about −5 dBm. Since the MNC describes the nondispersive NLS channel more accurately than the other two channels, this result suggests that the perturbative models are grossly inaccurate in the high-power regime.

## 5. Discussion

The capacity of three optical models, namely, the RPC, the LPC, and the MNC, were investigated. All three models are developed under two assumptions: channel memory is ignored and a sampling receiver is applied. Furthermore, two of these models, i.e., the RPC and the LPC, are based on perturbation theory and ignore signal–noise interaction, which makes them accurate only in the low-power regime. By tightly bounding the capacity of the RPC, by characterizing the capacity of the LPC, and by developing a tight upper bound on the capacity of the MNC, we showed that the capacity of these models, for the same underlying physical channel, behave very differently at high powers. Since the MNC is a more accurate channel model than the other two, one may conclude that the perturbative models become grossly inaccurate at high powers in terms of capacity calculation.

Note that the LPC model can be obtained from the MNC by neglecting the signal–noise interaction. Comparing the capacity of these two channels allows us to conclude that the impact of neglecting the signal–noise interaction on capacity is significant. Observe also that the capacity of the LPC model grows quickly with power because of the large capacity pre-log. Such a behavior is caused by the additive model used for the nonlinear distortion, which causes an artificial power increase at high SNR. A more accurate model than the RPC may be obtained by performing a normalization that conserves the signal power. Future work should consider more realistic channel models with nonzero dispersion and with more practical receivers.

## Figures and Tables

**Table 1:** Channel parameters.

Parameter	Symbol	Value
Attenuation	α	0.2dB/km
Nonlinearity	γ	$1.27\;{\left(W\cdot \mathrm{km}\right)}^{-1}$
Fiber length	*L*	5000km
Maximum bandwidth	$W_{N}$	125GHz
Emission factor	$n_{\mathrm{sp}}$	1
Photon energy	hν	$1.28\times 10^{-19}J$
Noise variance	$P_{N}$	−21.3dBm

## Appendix A. Proof of Theorem 1

The capacity of the regular perturbative channel can be written as

(A1) $$C_{\mathrm{RPC}}=\sup I\left(x;y\right),$$

where the supremum is over all the probability distributions on x that satisfy the power constraint (21). Let

(A2) $$w=x+{j\eta |x|}^{2}x.$$

We have that

(A3)Ix;y=hy−hy|x(A4)=hw+n−hw+n|x(A5)=hw+n−hn.

Using the entropy power inequality ([31], Sec. 2.2) and the Gaussian entropy formula ([34], Th. 8.4.1), we conclude that

(A6)hw+n≥log2hw+2hn(A7)=log2hw+πePN.

Substituting (A7) into (A5), and using again the Gaussian entropy formula ([34], Th. 8.4.1), we obtain

(A8) $$\begin{array}{c} I\left(x;y\right)\geq \log\left(2^{h\left(w\right)}+\pi eP_{N}\right)-\log\left(\pi eP_{N}\right). \end{array}$$

We take x circularly symmetric. It follows from (A2) that w is also circularly symmetric. Using ([35], Equation (320)) to compute h(w), we obtain

(A9) $$\begin{array}{c} h\left(w\right)=h\left({\left|w\right|}^{2}\right)+\log\pi . \end{array}$$

Substituting (A9) into (A8), we get

(A10) $$\begin{array}{c} I\left(x;y\right)\geq \log\left(\frac{2^{h\left({\left|w\right|}^{2}\right)}}{eP_{N}}+1\right). \end{array}$$

Next, to evaluate the right-hand side (RHS) of (A10), we choose the following distribution for the amplitude square $s={\left|x\right|}^{2}$ of x:

(A11) $$f_{s}\left(s\right)=\zeta \left(3\eta^{2}s^{2}+1\right)e^{-\lambda s},\;\;\;\;s\geq 0.$$

The parameters λ>0 and ζ>0 are chosen so that (A11) is a pdf and so that the power constraint (21) is satisfied. We prove in Appendix A.1 that by choosing these two parameters so that

(A12) $$\zeta =\frac{\lambda^{3}}{\lambda^{2}+6\eta^{2}}$$

and so that (24) holds, both constraints are met. In Appendix A.2, we then prove that

(A13) $$\begin{array}{c} h\left({\left|w\right|}^{2}\right)=-\log\zeta +\zeta \left(\frac{1}{\lambda }+\frac{18\eta^{2}}{\lambda^{3}}\right)\log e. \end{array}$$

Substituting (A13) and (A12) into (A10), we obtain (22). Although not necessary for the proof, in Appendix A.3, we justify the choice of the pdf in (A11) by showing that it maximizes h(w). (Based on (A5), the distribution that maximizes h(w) achieves capacity at high powers, where h(w+n)≈h(w).)

### Appendix A.1. Choosing ζ and λ

We choose the coefficients ζ and λ so that (A11) is a valid pdf and $E\left[s\right]\leq P$. Note that

(A14)∫0∞fs(s)ds=∫0∞ζ3η2s2+1e−λsds(A15)=ζλ2+6η2λ3.

Therefore, choosing ζ according to (A12) guarantees that $f_{s}\left(s\right)$ integrates to 1. We next compute $E\left[s\right]:$

(A16)Es=∫0∞sfs(s)ds(A17)=∫0∞sζ3η2s2+1e−λsds(A18)=ζ18η2λ4+1λ2.

Substituting (A12) into (A18), we obtain

(A19) $$\begin{array}{cc} E\left[s\right] & =\frac{18\eta^{2}+\lambda^{2}}{\lambda \left(\lambda^{2}+6\eta^{2}\right)}. \end{array}$$

We see now that imposing $E\left[s\right]\leq P$ is equivalent to (23). Observe that the RHS of (A19) and the objective function on the RHS of (22) are decreasing functions of λ. Therefore, setting the RHS of (A19) equal to *P*, which yields (24), maximizes the objective function in (22).

Finally, we prove that (24) has a single positive root. We have

(A20)f(λ)=Pλ3−λ2+6Pη2λ−18η2(A21)=λ2+6η2Pλ−1−12η2.

Note that f(λ)→∞ as λ→∞ and that the RHS of (A21) is negative when λ<1/P. Furthermore, f(λ) is monotonically increasing in the interval [1/P,∞). Indeed, when λ≥1/P,

(A22)ddλf(λ)=3Pλ2−2λ+6Pη2(A23)≥3λ−2λ+6Pη2(A24)>0.

This yields the desired result.

### Appendix A.2. Proof of (A13)

To compute the differential entropy of $t={\left|w\right|}^{2}$, we first determine the pdf of t. By definition,

(A25) $$t=s+\eta^{2}s^{3}.$$

Let now $g\left(x\right)=x+\eta^{2}x^{3}$. Since

(A26) $$\begin{array}{cc} \frac{\;d}{\;dx}g\left(x\right) & =1+3\eta^{2}x^{2}, \end{array}$$

we conclude that $g\left(x\right)$ is monotonically increasing for x≥0. Hence, g(x) is one-to-one when x≥0 and its inverse

(A27) $$q\left(x\right)=g^{-1}\left(x\right)$$

is well defined. Thus, the pdf of t is given by ([36], Ch. 5)

(A28)ft(t)=fsqtg′qt(A29)=ζ3η2q2t+1e−λqt3η2q2t+1(A30)=ζe−λq(t),t≥0.

Here, (A29) holds because of (A11). Using (A30), we can now compute $h\left(t\right)$ as

(A31)ht=−∫0∞ft(t)logft(t)dt(A32)=−logζ+λζloge∫0∞qte−λqtdt(A33)=−logζ+λζloge∫0∞re−λr1+3η2r2dr(A34)=−logζ+λζ1λ2+18η2λ4loge,

where in (A33) we used the change of variables $r=q\left(t\right)$. This proves (A13).

### Appendix A.3. f s (s) Maximizes h(w)

We shall prove that the pdf $f_{s}\left(s\right)=\zeta \left(3\eta^{2}s^{2}+1\right)e^{-\lambda s},\;\;s\geq 0$, maximizes $h\left(w\right)$. It follows from (A9) that, to maximize $h\left(w\right)$, we need to maximize $h\left(t\right)$. We assume that the power constraint is fulfilled with equality, i.e., that

(A35) $$E\left[s\right]=\int_{0}^{\infty }sf_{s}\left(s\right)\;ds=P.$$

Using the change of variables s=q(t), where q(t) was defined in (A27), we obtain

(A36) $$\int_{0}^{\infty }q\left(t\right)f_{s}\left(q\left(t\right)\right)q^{\prime }\left(t\right)\;dt=P.$$

Substituting (A28) into (A36) and using that $q^{\prime }\left(t\right)=1/g^{\prime }\left(q\left(t\right)\right)$, we obtain

(A37) $$\int_{0}^{\infty }q\left(t\right)f_{t}\left(t\right)\;dt=P.$$

It follows now from ([34], Th. 12.1.1) that the pdf that maximizes h(t) is of the form $f_{t}\left(t\right)=e^{\lambda_{0}+\lambda_{1}q\left(t\right)},\;\;t\geq 0$, where $\lambda_{0}$ and $\lambda_{1}$ need to be chosen so that (A37) is satisfied and $f_{t}\left(t\right)$ integrates to one. Using (A28), we get

(A38)fs(s)=ft(g(s))g′(s)(A39)=1+3η2s2eλ0+λ1s,s≥0.

By setting $\zeta =e^{\lambda_{0}}$ and $\lambda =\lambda_{1}$, we obtain (A11).

## Appendix B. Proof of Theorem 2

Fix λ≥0. It follows from (20) and (21) that

(A40) $$\begin{array}{cc} C_{\mathrm{MNC}}\left(P\right) & \leq \sup\left\{I\left(x;y\right)+\lambda \left(1-\frac{E\left[{\left|x\right|}^{2}\right]+P_{N}}{P+P_{N}}\right)\right\}, \end{array}$$

where the supremum is over the set of probability distributions that satisfy the power constraint (21). Next, we upper-bound the mutual information $I\left(x;y\right)$ as

(A41)Ix;y=hy−hy|x(A42)=hy−hn(A43)=h|y|+hy||y|+Elog|y|−hn(A44)=h|y|2+hy||y|−log2−hn(A45)≤h|y|2+logπ−hn(A46)=h|y|2−logePN,

where in (A43) we used ([35], Lemma 6.16) and in (A44) we used ([35], Lemma 6.15). We fix now an arbitrary input pdf $f_{x}\left(\cdot \right)$ that satisfies the power constraint and define the random variables $v={\left|y\right|}^{2}$ and $w=x+{j\eta |x|}^{2}x$. Next, we shall obtain an upper bound on $h\left(v\right)$ that is valid for all $f_{x}\left(\cdot \right)$. Let

(A47) $${\tilde{f}}_{v}\left(v\right)=\kappa e^{-\mu q\left(v\right)},\;\;\;v\geq 0$$

for some parameters κ>0 and μ>0. The function q(·) is defined in (A27). We next choose κ so that ${\tilde{f}}_{v}\left(v\right)$ is a valid pdf. To do so, we set $z=q\left(v\right)$, which implies that g(z)=v, and that

(A48) $$\left(1+3\eta^{2}z^{2}\right)\;dz=\;dv.$$

Therefore, integrating $\tilde{f}\left(v\right)$ in (A47), we obtain

(A49)∫0∞κe−μqvdv=κ∫0∞e−μz1+3η2z2dz(A50)=κμ2+6η2μ3.

We see from (A50) that the choice

(A51) $$\kappa =\frac{\mu^{3}}{\mu^{2}+6\eta^{2}}$$

makes ${\tilde{f}}_{v}\left(v\right)$ a valid pdf. Using the definition of the relative entropy, we have

(A52)Dfv(v)||f˜v(v)=∫−∞+∞fv(v)logfv(v)f˜v(v)dv(A53)=−h(v)−Elogf˜v(v).

Since the relative entropy is nonnegative ([34], Thm. 8.6.1), we obtain

(A54)hv≤−Evlogf˜v(v)(A55)=−logκ+μEqvloge.

Substituting (A46) and (A55) into (A40), we obtain

(A56)CMNC(P)≤−logePN−logκ+λ+supμEqvloge−λE|x|2+PNP+PN(A57)≤−logePN−logκ+λ+maxs>0μEqv||x|2=sloge−λs+PNP+PN.

The final upper bound (25) is obtained by minimizing (A57) over all λ≥0 and μ≥0.

## Appendix C. Proof of Theorem 3

It follows from (A55) and (A46) that

(A58) $$\begin{array}{cc} I\left(x;y\right) & \leq -\log\left(\kappa \right)+\mu E\left[q\left(v\right)\right]\log e-\log\left(eP_{N}\right), \end{array}$$

where $v={\left|y\right|}^{2}$. Moreover,

(A59)Eqv=Eq|w+n|2(A60)=Eq|w|2+|n|2+2Rwn*.

Next, we analyze the function q(x). We have

(A61)q′x=1g′qx(A62)=11+3η2q2x.

Furthermore,

(A63) $$\begin{array}{cc} q^{\prime \prime }\left(x\right) & =-\frac{6\eta^{2}q\left(x\right)}{{\left(1+3\eta^{2}q^{2}\left(x\right)\right)}^{3}}\leq 0. \end{array}$$

Therefore, $q\left(x\right)$ is a nonnegative concave function on [0,∞). Thus, for every real numbers x≥0 and y≥−x,

(A64) $$\begin{array}{cc} q\left(x+y\right) & \leq q\left(x\right)+q^{\prime }\left(x\right)y. \end{array}$$

Using (A64) in (A60), with $x={\left|w\right|}^{2}+{\left|n\right|}^{2}$ and $y=2R\left(wn^{*}\right)$, we get

(A65) $$\begin{array}{cc} E\left[q\left(v\right)\right] & \leq E\left[q\left({\left|w\right|}^{2}+{\left|n\right|}^{2}\right)+2q^{\prime }\left({\left|w\right|}^{2}+{\left|n\right|}^{2}\right)R\left(wn^{*}\right)\right]. \end{array}$$

Using (A64) once more with $x={\left|w\right|}^{2}$ and $y={\left|n\right|}^{2}$, we obtain

(A66)Eqv≤Eq|w|2+q′|w|2|n|2+2q′|w|2+|n|2Rwn*(A67)=Eq|w|2+PNEq′|w|2+2Eq′|w|2+|n|2Rwn*.

We shall now bound each expectation in (A67) separately. Since ${\left|w\right|}^{2}=g\left({\left|x\right|}^{2}\right)$, we have that

(A68)Eq|w|2=E|x|2(A69)≤P,

where the last inequality follows from (21). It also follows from (A62) that

(A70) $$\begin{array}{c} q^{\prime }{\left(|w\right|}^{2})\leq 1. \end{array}$$

Furthermore, (A62) and (A63) imply that the function $q^{\prime }\left(x\right)$ is positive and decreasing in the interval x≥0. Therefore,

(A71)Eq′|w|2+|n|2Rwn*≤Eq′|w|2+|n|2|R(wn*)|(A72)≤Eq′|w|2|R(wn*)|(A73)≤Eq′|w|2|w|·|n|(A74)=πPN2Eq′|w|2|w|(A75)≤πPN2maxt≥0tq′t2(A76)=πPN2maxt≥0t1+3η2q2t2,

where (A74) holds because $E\left[|n|\right]=\sqrt{\pi P_{N}/4}$ and the last equality follows from (A62). To calculate the maximum in (A76), we use the change of variables $t^{2}=x+\eta^{2}x^{3}$ to obtain

(A77)maxt≥0t1+3η2q2t2=maxx≥0x+η2x31+3η2x2(A78)=1123/83−1η,

where the last step follows by some standard algebraic manipulations that involve finding the roots of the derivative of the objective function on the RHS of (A77). Substituting (A78) into (A76), we obtain

(A79) $$\begin{array}{cc} E\left[q^{\prime }\left({\left|w\right|}^{2}+{\left|n\right|}^{2}\right)R\left(wn^{*}\right)\right] & \leq \frac{\sqrt{\pi P_{N}}}{2\times 12^{3/8}\sqrt{\left(\sqrt{3}-1\right)\eta }}. \end{array}$$

Substituting (A69), (A70), and (A79) into (A67), and the result into (A58), we obtain

(A80) $$\begin{array}{cc} I\left(x;y\right) & \leq -\log\kappa +\mu \left(\log e\right)\left\{P+P_{N}+\frac{\sqrt{\pi P_{N}}}{12^{3/8}\sqrt{\left(\sqrt{3}-1\right)\eta }}\right\}-\log\left(eP_{N}\right). \end{array}$$

Finally, we obtain (25) by substituting (A51) into (A80). Since the upper bound (A80) on mutual information holds for every input distribution that satisfies the power constraint, it is also an upper bound on capacity for every μ>0. To find the optimal μ, we need to minimize

(A81) $$\begin{array}{c} \log\left(\frac{\mu^{2}+6\eta^{2}}{\mu^{3}}\right)+\mu \left(P+B\right)\log e=\log\left(\frac{\exp\left(\mu \left(P+B\right)\right)\left(\mu^{2}+6\eta^{2}\right)}{\mu^{3}}\right), \end{array}$$

where *B* was defined in (28). Observe now that the function inside logarithm on the RHS of (A81) goes to infinity when μ→0 and when μ→∞. Therefore, since this function is positive, it must have a minimum in the interval [0,∞). To find this minimum, we set its derivative equal to zero and get (28). Note finally that, since (24) has exactly one real root, which was proved in Appendix A.1, (28) also has exactly one real root.

## Appendix D. Proof of Theorem 5

The proof uses similar steps as in ([37], Sec. III-C). We upper-bound the mutual information between the x and y expressed in polar coordinates as

(A82)I(x;y)=I(r0,θ0;r,θ)(A83)=I(r0,θ0;r)+I(r0,θ0;θ|r)(A84)=h(r)−h(r|r0,θ0)+h(θ|r)−h(θ|r,r0,θ0)(A85)≤h(r2)−E[log(r)]−log2−h(r|r0,θ0)+log(2π)−h(θ|r,r0,θ0).

In (A85), we used ([35], Equation (317)) and that h(θ|r)≤log(2π). Let now ${\tilde{f}}_{r^{2}}\left(\cdot \right)$ denote an arbitrary pdf for $r^{2}$. Following the same calculations as in (A52)–(A54), we obtain

(A86) $$\begin{array}{c} h\left(r^{2}\right)\leq -E_{r^{2}}\left[\log({\tilde{f}}_{r^{2}}\left(r^{2}\right))\right]. \end{array}$$

We shall take ${\tilde{f}}_{r^{2}}\left(\cdot \right)$ to be a Gamma distribution with parameters α>0 and $\beta =(P+P_{N})/\alpha$, i.e.,

(A87) $$\begin{array}{c} {\tilde{f}}_{r^{2}}\left(z\right)=\frac{z^{\alpha -1}e^{-z/\beta }}{\beta^{\alpha }\Gamma \left(\alpha \right)},\;z\geq 0. \end{array}$$

Here, Γ(·) denotes the Gamma function. Substituting (A87) into (A86), we obtain

(A88)E[log(f˜r2(r2))](A89)=2(α−1)Elog(r)−αEr02+PNP+PNloge−αlogP+PNα−log(Γ(α)).

It follows from (15) that the random variables r and $\theta_{0}$ are conditionally independent given $r_{0}$. Therefore,

(A90) $$h\left(r|r_{0},\theta_{0}\right)=h\left(r|r_{0}\right).$$

Next, we study the term $h(\theta |r,r_{0},\theta_{0})$ in (A85). From Bayes’ theorem and (15) it follows that, for every $\theta^{\prime }\in \left[0,2\pi \right),$

(A91) $$f_{\theta |r,r_{0},\theta_{0}}\left(\theta |r,r_{0},\theta_{0}\right)=f_{\theta |r,r_{0},\theta_{0}}\left(\theta -\theta^{\prime }|r,r_{0},\theta_{0}-\theta^{\prime }\right).$$

Therefore,

(A92)h(θ|r,r0,θ0)=∫02πfθ0(θ0)h(θ|r,r0,θ0=θ0)dθ0(A93)=∫02πfθ0(θ0)h(θ−θ0|r,r0,θ0=0)dθ0(A94)=∫02πfθ0(θ0)h(θ|r0,θ0=0)dθ0(A95)=h(θ|r,r0,θ0=0).

Here, (A94) follows because differential entropy is invariant to translations ([34], Th. 8.6.3). Substituting (A89), (A86), (A90), and (A95) into (A85), we obtain

$$\begin{array}{cc} I(r_{0},\theta_{0};r,\theta ) & \leq \alpha \log\left(\frac{P+P_{N}}{\alpha }\right)+\log\left(\Gamma (\alpha )\right)+\log\left(\pi \right)+\alpha \frac{E\left[r_{0}^{2}\right]+P_{N}}{P+P_{N}}\log e \end{array}$$

(A96) $$\begin{array}{cc} & \;+\left(1-2\alpha \right)E\left[\log(r)\right]-h\left(r|r_{0}\right)-h\left(\theta |r,r_{0},\theta_{0}=0\right). \end{array}$$

Fix λ≥0. We next upper bound $C_{\mathrm{MNC}}$ using (A96) as

(A97)CMNC(P)≤supI(r0,θ0;r,θ)+λ1−E[r02]+PNP+PN≤αlogP+PNα+logΓ(α)+log(π)+λ+sup{(αloge−λ)Er02+PNP+PN+(1−2α)Elog(r)−hr|r0(A98)−h(θ|r,r0,θ0=0)},

where the supremum is over the set of input probability distributions that satisfy (21). We complete the proof by noting that the supremum in (A98) is less than or equal to $\max_{r_{0}>0}\left\{g_{\lambda ,\alpha }\left(r_{0},P\right)\right\}$, where $g_{\lambda ,\alpha }\left(r_{0},P\right)$ is defined in (36).
